# Supplementary material for: Splice-Junction-Based Mapping of Alternative Isoforms in the Human Proteome
Source: Cell Rep. Author manuscript; Available in PMC 2020 Jan 15. (PMC6961840; doi:10.1016/j.celrep.2019.11.026)
Supplement: 2 [file NIHMS1546469-supplement-2.zip › DF1/PXD010154/Ovary/Ovary_2_MAP4_AGQNDPLKDSYVPLELAK.pdf]

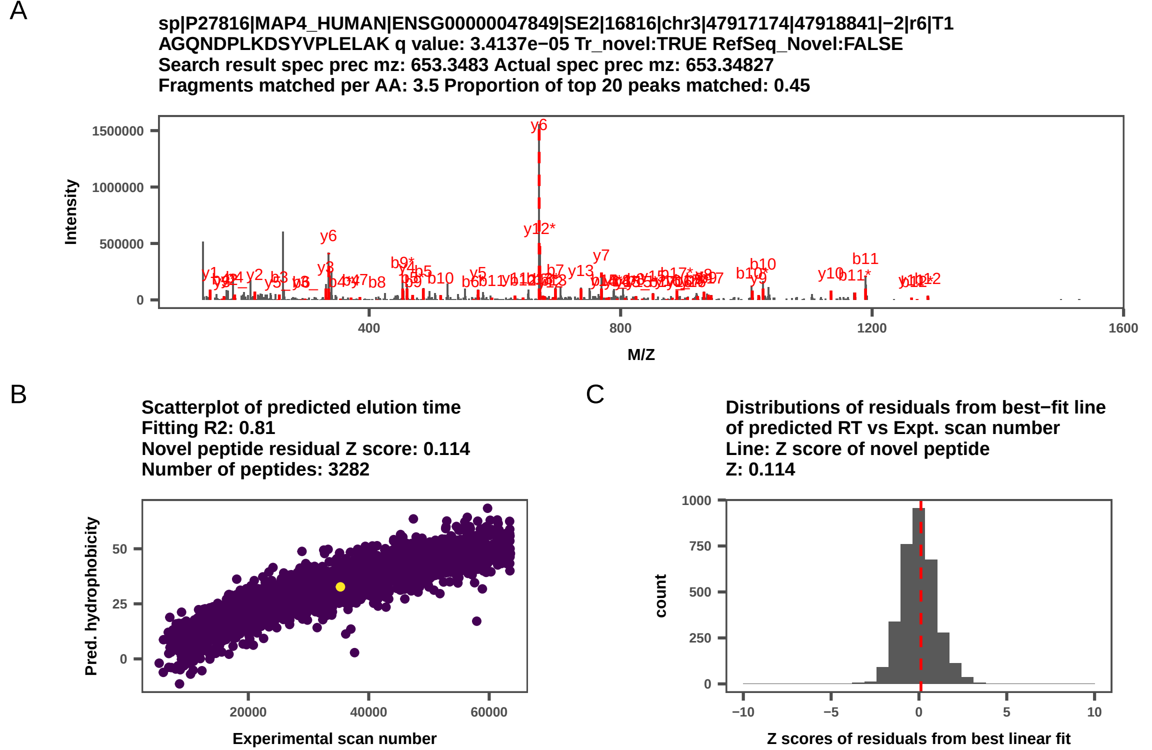
A
sp|P27816|MAP4_HUMAN|ENSG00000047849|SE2|16816|chr3|47917174|47918841|−2|r6|T1
AGQNDPLKDSYVPLELAK q value: 3.4137e−05 Tr_novel:TRUE RefSeq_Novel:FALSE
Search result spec prec mz: 653.3483 Actual spec prec mz: 653.34827
Fragments matched per AA: 3.5 Proportion of top 20 peaks matched: 0.45
Intensity
1500000
1000000
500000
0
M/Z
400
800
1200
1600
y6
y12*
y7
b10
b11
y10
b11*
B
Scatterplot of predicted elution time
Fitting R2: 0.81
Novel peptide residual Z score: 0.114
Number of peptides: 3282
Pred. hydrophobicity
50
25
0
Experimental scan number
20000
40000
60000
C
Distributions of residuals from best−fit line
of predicted RT vs Expt. scan number
Line: Z score of novel peptide
Z: 0.114
count
1000
750
500
250
0
Z scores of residuals from best linear fit
−10
−5
0
5
10
